# Effects of Thermal Cycles on Mechanical Properties of RPECC: Static and Dynamic Compressive Performance

**DOI:** 10.3390/ma18122846

**Published:** 2025-06-17

**Authors:** Shaohua He, Zhiliang Chen, Changxi Liu, Jincai Chen, Huanwei Chen, Zhitao Yu

**Affiliations:** 1 School of Civil and Transportation Engineering, Guangdong University of Technology, Guangzhou 510006, China2112309021@mail2.gdut.edu.cn (Z.C.); 2112309132@mail2.gdut.edu.cn (C.L.); yuzt@gdut.edu.cn (Z.Y.); 2 Shanzhan Branch of Guangdong Road & Bridge Construction Development Co., Ltd., Meizhou 514000, China; 2112109007@mail2.gdut.edu.cn

**Keywords:** rubberized concrete, engineered cementitious composites, thermal cycling, compressive properties, dynamic increase factor

## Abstract

This study explores the impact of thermal cycling and rubber particle content on the static and dynamic compressive properties of rubber–polyethylene fiber-reinforced engineered cementitious composites (RPECC). Through static and dynamic compression tests, supplemented by scanning electron microscopy and energy-dispersive X-ray spectroscopy, the mechanical behavior and microstructural evolution of RPECC under thermal cycling were analyzed. Results indicate that increasing rubber content from 10% to 30% enhances toughness and strain capacity but reduces the static compressive strength of ECC by up to 17.9% at 30%. Thermal cycling reduced strength: static and dynamic compressive strengths decreased by 18.0% and 41.2%, respectively, after 270 cycles. Dynamic tests demonstrated that RPECC is sensitive to strain rate. For example, C-20 specimens exhibited increases in dynamic strength of 6.9% and 9.9% as strain rate rose from 60.2 s^−1^ to 77.4 s^−1^ and 110.8 s^−1^, respectively, and the dynamic increase factor correlated linearly with strain rate. By contrast, excessive rubber content (30%) diminishes dynamic strengthening, indicating that 20% rubber is optimal for enhancing strain rate sensitivity. Thermal cycling facilitates the formation of hydration products, such as calcium hydroxide, and creates interfacial defects, further deteriorating mechanical performance. These findings provide a reliable foundation for optimizing RPECC mix design and ductility in environments subject to temperature fluctuations and dynamic loading.

## 1. Introduction

Engineered cementitious composites (ECC) have garnered significant attention in structural engineering due to their exceptional tensile strain-hardening behavior, effective crack control, and self-healing properties, which make them particularly suitable for bridge applications [1,2,3,4,5]. Under normal environmental conditions, ECC demonstrates remarkable toughness, ductility, and durability, thereby substantially enhancing the performance and longevity of structures. Nevertheless, applying ECC in harsh environments, especially those characterized by thermal cycling and dynamic loading, poses considerable challenges. Thermal cycling, encompassing diurnal temperature variations and seasonal changes, induces thermal stresses, microcrack propagation, and instability in hydration products. These challenges are particularly pronounced in ECC link slabs of jointless bridges, where the slab experiences significant cyclic thermal stresses due to solar radiation and environmental temperature fluctuations. Previous studies have documented compressive strength reductions of up to 27% in ECC after a mere 30 thermal cycles were subjected to temperatures of 80 °C [6], underscoring the critical necessity for examining the thermal resilience of ECC materials.

The performance of ECC under dynamic loading, such as vehicle impact or seismic events, is another critical concern. While ECC demonstrates exceptional energy absorption and crack resistance under static conditions, its behavior under high strain rates is poorly understood. Dynamic loading can significantly influence the mechanical properties of ECC, particularly in thermally cycled environments. Research indicates that the dynamic peak stress of ECC can decrease by as much as 60% under temperature cycles ranging from 400 °C to 800 °C, concurrently with an increase in critical strain from 0.9% to 2.4% [7]. Similar to the findings of Ahmad et al. [8], such extreme, high-temperature cycles (400–800 °C) primarily induce phase decomposition (e.g., dehydration of calcium silicate hydrate (C-S-H) gel) and severe microcracking, distinct from the microstructural evolution under mild thermal cycles (20–80 °C). For the latter, moderate temperature elevation (80 °C) accelerates cement hydration, promoting the formation of hydration products such as calcium hydroxide (CH). This process, though beneficial for early strength development, may introduce weak crystalline phases over repeated cycles, thereby affecting long-term mechanical performance. These observations highlight the susceptibility of ECC to the combined effects of thermal and dynamic loading, which may accelerate ageing, diminish durability, and compromise structural integrity. Incorporating fibers to enhance ECC’s dynamic properties leads to microstructural alterations, including changes in pore structures and interfacial transition zones. During thermal cycling, these modifications can disrupt moisture diffusion and hydration processes, forming hydration by-products such as calcium hydroxide and weakening the bond between the fibers and the matrix. Such degradation mechanisms further diminish ECC’s compressive strength and overall performance in aggressive environmental conditions.

Many scholars have explored incorporating rubber particles into ECC to address these challenges, forming rubber–polyethylene fiber-reinforced engineered cementitious composites (RPECC). Rubber particles offer several potential benefits, including improved thermal insulation, reduced moisture permeability, and enhanced energy absorption [9]. While rubber waste has emerged as a promising additive, other wastes have also been used for concrete modification. For example, electronic waste (e-waste) such as glass fibers from crushed circuit boards or liquid crystal panels can enhance the thermal conductivity and durability of concrete but tends to weaken the mechanical properties and has poor interfacial bonding due to its smooth texture [10]. In contrast, rubber particles derived from waste tires have significant advantages in thermally fluctuating environments. The elastic deformation ability of rubber provides additional stress dispersion and deformation buffering during thermal cycles, which can mitigate the adverse effects of thermal expansion and contraction. Studies have demonstrated that adding rubber particles can significantly enhance the ductility of ECC [11], reducing crack width and improving flexural performance. For instance, smaller-sized rubber particles (e.g., 80 CR) have been proven to improve the flexural deformation of ECC more effectively than larger particulates [12]. Moreover, the hydrophobic nature of rubber reduces moisture penetration, further enhancing the durability of ECC materials in humid or wet environments, making it a promising candidate for applications in bridges often exposed to fluctuating temperatures and dynamic loads.

However, the incorporation of rubber particles also introduces new challenges. The primary issue is the inherent low stiffness of rubber, which results in weaker bonding with the cement matrix and increased porosity. This leads to a reduction in compressive strength, with studies reporting decreases of up to 30% when rubber content reaches 30.2% of the total aggregate volume [13]. Additionally, the presence of rubber particles can induce interfacial defects and microstructural inhomogeneities, further compromising the mechanical performance of RPECC [14]. These drawbacks are particularly pronounced under thermal cycling conditions, where repeated heating and cooling cycles can amplify the degradation of the rubber matrix interface and promote microcrack formation. Despite these challenges, the potential benefits of RPECC in harsh environments need further investigation, particularly regarding its performance under combined thermal and dynamic loading.

This study builds on our previous work (He et al., 2025) [2], which focused on the static and dynamic splitting tensile properties of RPECC under thermal cycles (20–80 °C). Previous studies have shown that rubber particles enhanced the tensile ductility and deformability of RPECC, but the tensile strength was reduced by 14.1–20.6% due to thermal cycling that disrupted the interfacial transition zone between the different material phases within RPECC. However, it left unresolved questions regarding the compressive behavior of RPECC—critical for applications such as bridge link slabs, where compressive stresses dominate under traffic loading and temperature rise.

This study aims to bridge these knowledge gaps by evaluating the static and dynamic compressive properties of RPECC under thermal cycling conditions. Through a combination of static and dynamic compression tests, as well as advanced microstructural characterization techniques such as scanning electron microscopy (SEM) and energy-dispersive X-ray spectroscopy (EDS), the effects of rubber content (10%, 20%, 30%), strain rates, and thermal cycles (90, 180, 270) on the mechanical behavior of RPECC are investigated. The mechanisms of performance degradation, including interfacial defects, hydration product formation, and pore coarsening, are also discussed.

## 2. Experiment Program

### 2.1. Mix Proportions

This study investigated the impact of rubber particles on the compressive properties of rubber particle-reinforced engineered cementitious composite (RPECC) by employing three specific volume replacement ratios: 10%, 20%, and 30%. Table 1 summarizes the mixture proportions for all RPECC subsets. Previous studies have investigated the basic mechanical properties of PE-ECC containing rubber powder [15].

**Figure 1:**
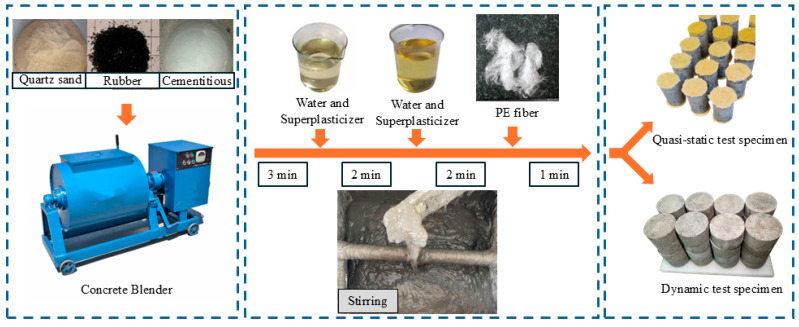
Preparation of RPECC.

The raw materials used included P-O 52.5 cement, granulated blast furnace slag powder of S105 grade, Class F-Grade I fly ash, high-strength polyethylene (PE) fibers, 0.3 mm rubber particles, purchased from the Shiyun Mineral Products Processing Plant (Lingshou County, China) and quartz sand, with particle sizes ranging from 0.15 mm to 0.25 mm. More detailed information on fibers is provided in Table 2. The supplier’s production process for rubber particles begins with comprehensive crushing, where scrap tires (with bead wires removed) are crushed into rubber blocks using a multi-functional crusher. Subsequently, medium crushing reduces these rubber blocks into 10–20 mm granules via an ambient-temperature rubber medium crusher. The process then moves to fine crushing, where the 10–20 mm granules are further processed in a precision grinder to produce two distinct fractions: 1–3 mm and 3–6 mm rubber particles. This stage simultaneously separates the rubber from the nylon fibers. Following this, a vibration-based fiber separator removes the nylon fibers to ensure the purity of the rubber granules. Optionally, for applications requiring finer material, the 1–3 mm particles can undergo fine milling in a powder mill to produce rubber powder ranging from 0.6 mm down to 0.09 mm. Specifically, the 0.3 mm rubber particles utilized in this study correspond to the 1–3 mm fraction generated during the fine crushing step (Step 3), as illustrated in Figure 1 (rubber powder material image).

The PE fibers used in this study were high modulus fibers sold in Guangzhou City, Guangdong Province, China. Additionally, a polycarboxylate-based high-efficiency water-reducing agent was employed, which has a 40% water-reduction capability.

### 2.2. Specimen Preparation and Maintenance

As shown in Figure 1, the mixing process of RPECC involves several steps to ensure a well-combined and high-quality final product. Initially, dry mixing of cement, slag, fly ash, other cementitious materials, rubbers, and sands occurs for 3 min to achieve an even dispersion of the rubber and sand. Following this, 50% of a mixed solution containing water and a water-reducing agent is gradually added to the dry ingredients and stirred for 2 min, promoting the hydration reaction of the cementitious materials. The remaining solution is then added while rapidly stirring until a uniform slurry is formed. Once uniformity is achieved, pre-dispersed PE fibers are introduced and mixed thoroughly to ensure even distribution. The final RPECC slurry is poured into test molds, and the RPECC specimens are cured in a standard environment at 95% relative humidity for 28 days to guarantee complete hydration and the development of desired mechanical properties.

The thermal cycling tests for the prepared specimens began 28 days after curing and were designed to simulate the rapid temperature increases of bridge decks in South China during summer, with temperatures ranging from 20 °C to 80 °C. The number of cycles was set at 90, 180, or 270. Each cycle comprised a 2-h low-temperature period at 20 °C, followed by a gradual increase to 80 °C at 1.2 °C/min over approximately 50 min, maintained for 2 h. The cooling back to 20 °C also took around 50 min, resulting in a total cycle time of about 6 h. Each condition, including those with no thermal cycles (0), was tested with three specimens, and the results were averaged for discussion. Although mass loss or permeability changes were not quantitatively monitored between cycles, post-test scanning electron microscopy (SEM) analysis (Section 3) provided qualitative evidence of internal microcracks and interfacial defects induced by thermal fatigue, such as debonding at rubber–cement interfaces and coarsened pores.

### 2.3. Test Methods

#### 2.3.1. Static Compression Test

**Figure 2:**
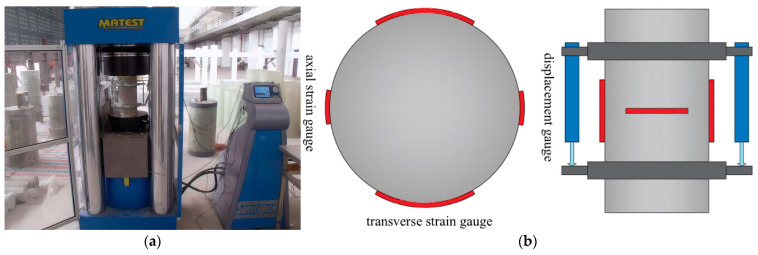
Static compression test setup: (**a**) Device; (**b**) Arrangement of strain gauges and sensors.

The static compressive properties of the RPECC were tested using standard cylinders with a diameter of 100 mm and a height of 200 mm. As shown in Figure 2a, a MATEST C088-01 material compression testing machine was utilized to conduct the compression tests, with the compressive strength determined according to the JGJ/T70-2009 standard [16] and the elastic modulus measured following ASTM guidelines C09 [17]. According to this standard, the modulus of elasticity is given by Equation (1). Four strain gauges were attached around the circumference of each cylinder to measure both longitudinal and transverse strains, while two displacement gauges, positioned 180° apart on the cylindrical fixture, measured axial deformation to facilitate the calculation of compressive strain. Since the strain gauges may be damaged when concrete cracks occur, the displacement gauges serve as an alternative measurement method. Figure 2b shows the layout of the strain gauges.

(1) $$E=\frac{S_{2}-S_{1}}{\varepsilon_{2}-0.000050}$$

where S_2_ is 40% of the peak stress, ε_2_ is the longitudinal strain corresponding to S_2_, and S_1_ is the stress corresponding to a longitudinal strain of 0.000050.

The compression tests were conducted at a loading rate of 0.18 mm/min, corresponding to a strain rate of approximately 1 × 10^−5^ s^−1^ [18,19]. A multi-channel static data collector (TDS-530) was synchronized with the MATEST testing machine to record axial load and displacement. Concurrently, environmental parameters such as ambient temperature and humidity were also monitored during the tests to aid in correcting and analyzing the results. Using the collected data, stress–strain curves were generated, and appropriate formulas were applied to determine the compressive strength and elastic modulus of the RPECC.

#### 2.3.2. Dynamic Compression Test

**Figure 3:**
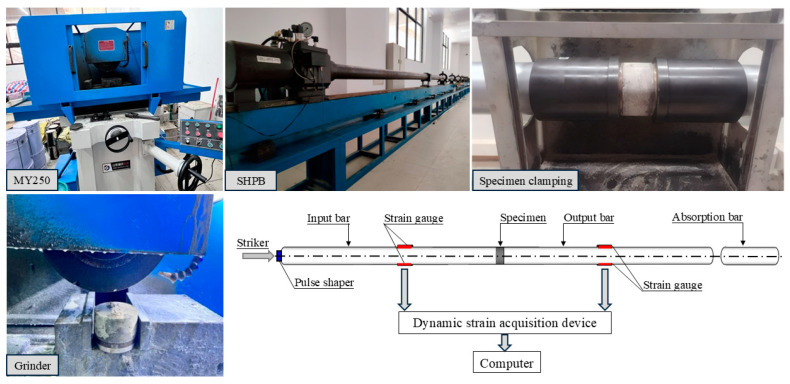
Grind device and SHPB system.

Figure 3 presents the devices used in the dynamic compression tests. The specimen, shaped like a cake, has a diameter of 100 mm and a height of 50 mm. Before conducting the tests, all specimens were ground using a high-precision grinder (MY250), which offers a parallelism accuracy of up to 0.01 mm over 300 mm and a surface smoothness accuracy of Ra 0.1 µm. The grinding depth was set to 0.76 mm to ensure that the flatness of each sample remained within 0.02 mm, thereby minimizing experimental errors. The dynamic loading was performed using the Split Hopkinson Pressure Bar (SHPB) system, which consists of an impact bar, an incident bar, a transmission bar, an energy-absorbing bar, and a data acquisition system.

During the test, the control air pressures of the Split Hopkinson Pressure Bar (SHPB) system were set to 0.6 MPa, 0.7 MPa, and 0.8 MPa. These pressures corresponded to strain rates of 37 s^−1^, 53 s^−1^, and 66 s^−1^, respectively. Throughout the tests, three types of pulses were recorded: the incident pulse ε_i_(t), the transmitted pulse ε_t_(t), and the reflected pulse ε_r_(t). The dynamic compression test was designed to align with the principles of one-dimensional stress wave theory [20,21] and the assumption of stress uniformity within the specimen. The stress σ_s_(t), strain ε_s_, and strain rate $\dot{\varepsilon }$_s_ were calculated using the following formulas [22]:

(2) $$\sigma_{s}\left(t\right)=\frac{{\mathrm{AE}}_{0}}{A_{s}}\varepsilon_{t}\left(t\right)$$

(3) $$\varepsilon_{s}=-\frac{{2c}_{0}}{l_{0}}\int_{0}^{t}\varepsilon_{r}\mathrm{dt}$$

(4) $${\dot{\varepsilon }}_{s}=-\frac{{2c}_{0}}{l_{0}}\varepsilon_{r}$$

where A is the cross-sectional area of the rod, E_0_ is the modulus of elasticity, c_0_ is the wave velocity, A_s_ denotes the cross-sectional area of the specimen, l_0_ denotes the length of the specimen, and t denotes time. To ensure stress uniformity within the specimen and validity of the one-dimensional stress wave assumption, pulse shaping techniques were applied using copper shims. Incident and transmitted pulses were monitored to confirm equilibrium prior to peak stress, as required by SHPB theory [20,21]. Tests violating this condition were discarded.

#### 2.3.3. Scanning Electron Microscopy

The SEM analysis was performed using an S-3400N-II scanning electron microscope to observe RPECC specimens. Before SEM analysis, the RPECC specimen underwent a uniform gold film coating to enhance conductivity, thereby ensuring smooth electron scanning and acquiring high-quality images. The observation focused on three key aspects: first, the interaction between PE fibers and the paste matrix, essential for elucidating the reinforcement mechanisms and mechanical properties of RPECC materials; second, the development of microcracks, as their initiation and propagation are closely associated with the durability and failure behavior of the composite; and third, the degree of hydration within the RPECC cementitious matrix, which directly influences material performance. These focal points provide in-depth insights into the microstructural characteristics of RPECC and their subsequent effects on macroscopic properties.

## 3. Results and Discussion

### 3.1. Quasi-Static Compressive Behavior

#### 3.1.1. Failure Pattern of Static Compressive Samples

**Figure 4:**
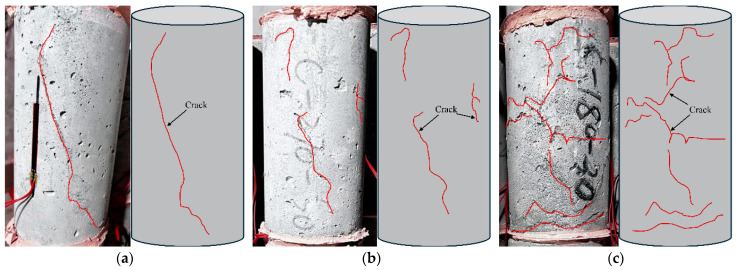
Failure mode of quasi-static compression specimen: (**a**) C-10; (**b**) C-20; (**c**) C-30.

In this study, “quasi-static compression” refers to compression tests with a strain rate of about 1 × 10^−5^ s^−1^, which is between strictly static and dynamic, and is closer to the actual scenario of slow loading in engineering. Under quasi-static compression conditions, the RPECC cylinders prepared in this study demonstrate distinct regularities and characteristics in their failure modes. The damage of the naturally cured RPECC primarily features longitudinal cracks extending from the top to the bottom of the specimen surface, accompanied by numerous fine microcracks. An increase in rubber content leads to a denser and more uniform microcrack network, resembling the failure characteristics of ECC previously reported [23]. For the thermal fatigue RPECC subjected to 90, 180, and 270 thermal cycles, only slight variations in failure modes are observed across different rubber contents, regardless of the number of thermal cycles applied, as illustrated by the representative specimen shown in Figure 4. It is worth noting that the diagonal crack damage in the C10 model may be caused by the non-parallelism of the pressure surfaces. The minimal influence of thermal cycles on the compression failure mode can be attributed to several factors. Firstly, rubbers’ excellent elasticity and flexibility effectively absorb and disperse forces during thermal cycling and static compression, minimizing damage to the cement matrix and preserving the failure mode. Secondly, the interfacial transition zone (ITZ) between the rubber and the cement matrix may change after thermal cycling, resulting in microcracks propagating and slipping at the interface. These contribute to a consistent failure mode, highlighting the critical impact of material composition and structure on the fatigue of RPECC under compression.

#### 3.1.2. Static Compressive Stress vs. Strain Relationship

**Figure 5:**
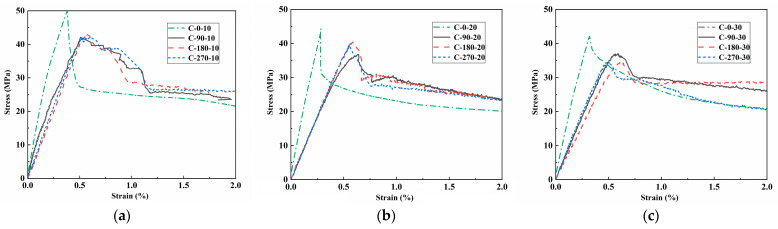

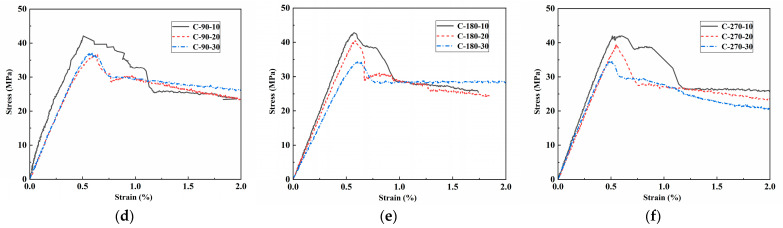
Comparisons of stress–strain curves: (**a**) Thermal effects on 10% rubber case; (**b**) Thermal effects on 20% rubber case; (**c**) Thermal effects on 30% rubber case; (**d**) Rubber effects on 90-cycle case; (**e**) Rubber effects on 180-cycle case; (**f**) Rubber effects on 270-cycle case.

Figure 5 depicts the compressive stress–strain behavior of RPECC under different thermal cycles and rubber contents. For specimens with 10% rubber, Figure 5a shows that thermal cycling significantly affects mechanical properties compared to the non-cycled sample (C-0-10), resulting in a 25–30% decline in peak stress and a 40–50% reduction in elastic modulus, as shown by decreased initial slopes. Peak strain increases by 60–80%, indicating enhanced deformation capacity in thermally cycled specimens. The post-peak response transitions from an abrupt stress drop in C-0-10 to a gradual 15–20% reduction in the thermally cycled samples, suggesting thermal-induced microcrack homogenization. Mechanical properties stabilize after 90 cycles, with less than 5% variation between C-90-10, C-180-10, and C-270-10, revealing a threshold-like degradation pattern. In specimens with 20% and 30% rubber content, similar trends show peak stresses reduced by 18–22% and 12–15%, and elastic modulus decreases of 30–35% and 25–28%, respectively, indicating that higher rubber content enhances strain accommodation and mitigates thermal damage.

Figure 5d–f illustrate the cross-comparisons of the stress–strain curves derived from varying rubber content under identical thermal cycles. The data indicates that, under the same thermal cycling conditions, a 10% increase in rubber content leads to a decrease in compressive strength by 12–15 MPa and a reduction in elastic modulus by 2–3 GPa, while the failure strain increases by 0.3–0.4%. Furthermore, post-peak ductility indices show an improvement of 25–30% for each 10% increment in rubber content, aligning with the findings of Khaloo et al. [24] regarding rubber-modified concrete without thermal history.

The coupled mechanisms underlying the observed properties involve degradation of the interfacial transition zone and interactions between the rubber and cement matrix. The process of thermal cycling induces differential expansion, with rubber exhibiting a coefficient of thermal expansion (CTE) of approximately 200 × 10^−6^/°C, in contrast to the cement matrix’s CTE of roughly 10 × 10^−6^/°C. This disparity generates microcracking that compromises the composite material’s stiffness and strength. Rubber particles, possessing a 5% to 10% lower density than the cement matrix, function as stress concentrators during thermal fluctuations while inhibiting crack propagation through absorbing energies. This dual effect explains the stabilized performance observed after 90 thermal cycles and the corresponding enhancement in ductility. RPECC with higher rubber content exhibits reduced thermal degradation, attributable to the viscoelastic properties of rubber, which dissipate thermal stresses more efficiently than traditional rigid aggregates. Ultimately, the significance of the particle-matrix interface characteristics in dictating mechanical response surpasses that of thermal history within the parameters tested.

#### 3.1.3. Quasi-Static Compressive Strength and Elastic Modulus

**Figure 6:**
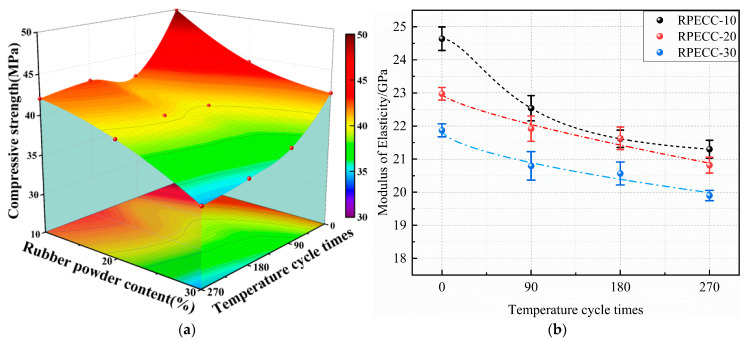
Influence of thermal cycle and rubber content on RPECC: (**a**) Compressive strength; (**b**) Elastic modulus.

Table 3 presents the quasi-static compressive strength and elastic modulus of RPECC subjected to varying thermal cycles (0, 90, 180, and 270) and rubber particle contents (10%, 20%, and 30%). As shown in Figure 6a, compressive strength displays a dual dependency on these parameters, achieving a maximum of 50.01 MPa at 10% rubber content and no thermal cycles while declining to a minimum of 34.52 MPa at 30% rubber content and 270 thermal cycles. Specifically, increasing rubber content from 10% to 30% without thermal cycles results in a compressive strength reduction of 11.3% (5.64 MPa) for 20% rubber and 15.8% (7.9 MPa) for 30%. This trend is consistent across all thermal cycle groups, with strength reductions ranging from 4.8% to 17.9% for every 10% increase in rubber. The mechanisms behind this decline include the low modulus of rubber particles, interfacial debonding between rubber and the cement matrix, and increased porosity from rubber incorporation [25]. Thermal cycles further degrade strength, but their impact lessens at higher rubber contents.

The elastic modulus follows similar degradation patterns, decreasing by 8.4–13.4% for 10% rubber and 6.7–11.1% for 0-cycle specimens as thermal cycles and rubber content increase (Figure 6b). This dual degradation aligns with compressive strength trends, confirming rubber’s role in reducing matrix stiffness and thermal cycling’s cumulative damage effect. Specimen C-0-10’s elastic modulus decreases by 8.4% (90 cycles), 12.2% (180 cycles), and 13.4% (270 cycles), while increasing rubber content from 10% to 30% under 0 cycles causes an 11.1% modulus reduction. The synergistic effect arises from thermal fatigue-induced microcrack propagation and rubber–cement interface deterioration, consistent with Zheng et al.’s findings on hybrid degradation mechanisms [26]. The nonlinear reduction trajectory implies threshold effects in thermal damage accumulation, particularly beyond 180 cycles, where modulus losses stabilize marginally.

Both compressive strength and elastic modulus degradation indicate the material’s trade-off between thermal stability and mechanical performance. While rubber addition mitigates thermal sensitivity by lowering thermal conductivity and raising volumetric heat capacity [27], its structural compromises dominate the mechanical response. Thermal cycles induce progressive but bounded damage, as evidenced by diminishing strength reduction amplitudes. This saturation effect suggests that microstructural damage reaches a pseudo-equilibrium state after repeated thermal loading. However, the non-monotonic relationship between rubber content and thermal degradation amplitude highlights complex interactions among pore structure, interfacial adhesion, and thermal stress redistribution. These findings emphasize the necessity for multi-parameter optimization when designing RPECC for thermo-mechanical environments.

### 3.2. Dynamic Compressive Behavior

#### 3.2.1. Failure Pattern Dynamic Compressive Samples

**Figure 7:**
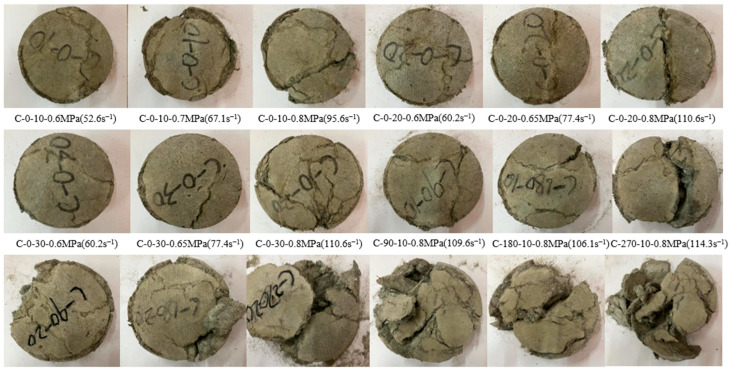
Typical impact failure modes of RPECC.

RPECC’s dynamic fracture pattern under impact loading varies with strain rate, rubber content, and thermal cycles, as illustrated in Figure 7. At lower strain rates (52.6 s^−1^, 0.6 MPa), specimens with 10% rubber content develop surface cracks while maintaining a relatively intact cylindrical shape. As the strain rate increases to 67.1 s^−1^ (0.7 MPa), the number and size of cracks grow, with microcracks becoming more pronounced at the edges. At the highest strain rate of 95.6 s^−1^ (0.8 MPa), severe damage is observed, characterized by large cracks and concrete spalling, particularly along the edges, although the cylindrical form is preserved. This trend is consistent across specimens with 20% and 30% rubber content, where increased strain rates intensify damage due to insufficient time for internal stress redistribution, leading to localized stress concentrations and material failure. These findings align with previous studies on rubberized concrete [28], emphasizing the critical role of strain rate in dynamic failure mechanisms.

Rubber content significantly impacts the dynamic fracture pattern of RPECC, particularly under high-strain-rate conditions (0.8 MPa). Specimens with 30% rubber content display finer and more densely distributed cracks than those with 10% and 20%, suggesting improved stress homogenization and crack resistance attributed to rubber’s energy-absorbing properties. However, introducing thermal cycling complicates the failure behavior; damage severity increases with the number of cycles, especially at higher rubber contents. After 270 thermal cycles, specimens with 10% rubber content exhibit noticeable splitting, while those with 30% rubber show complete deterioration, primarily due to thermal stress-induced microcracks and interfacial degradation between the rubber and cement matrix. This interaction amplifies initial defects [29] and diminishes bonding strength, exacerbating damage under dynamic loading conditions. The intricate interplay between material composition and environmental factors plays a crucial role in determining the dynamic mechanical performance of RPECC.

#### 3.2.2. Dynamic Compressive Stress vs. Strain Relationship

**Figure 8:**
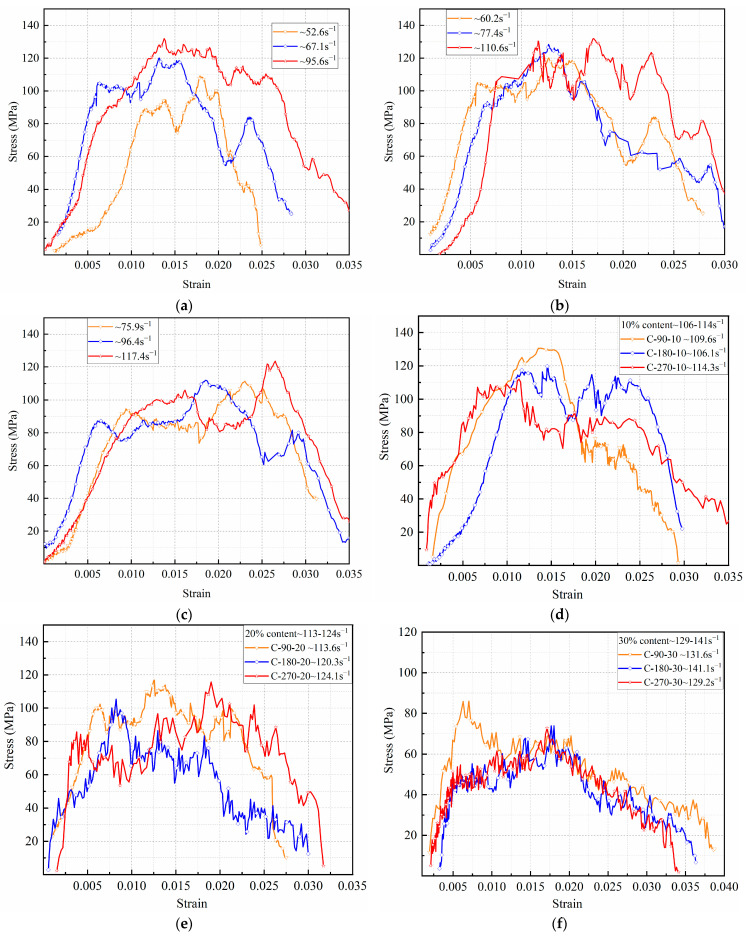
Dynamic stress–strain curves of RPECC under varying rubber contents and thermal cycles, showing plastic deformation, multi-peak characteristics due to fiber bridging, and reduced peak stress with higher rubber content: (**a**) 10% rubber (naturally cured); (**b**) 20% rubber (naturally cured); (**c**) 30% rubber (naturally cured); (**d**) 10% rubber (thermal cycled); (**e**) 20% rubber (thermal cycled); (**f**) 30% rubber (thermal cycled).

The dynamic stress–strain behavior of RPECC under impact loading demonstrates distinctive characteristics influenced by the rubbers and thermal cycling. As shown in Figure 8, dynamic curves reveal a pronounced plastic deformation stage followed by a rapid failure phase, differing from conventional trapezoidal stress–strain profiles, which typically include an elastic phase. The plastic phase is characterized by a “multi-peak” pattern, where the initial peak corresponds to the ultimate stress prior to concrete fragmentation. Subsequent peaks result from the bridging effect of PE fibers, which temporarily bear the stress until crack propagation exceeds their capacity. The concave regions between these peaks indicate rapid post-fragmentation expansion facilitated by rubber particles that accommodate deformation without imposing restrictions, thereby reducing stress levels. The dynamic failure stage exhibits a steeper slope than static compression, attributed to reduced dynamic friction between PE fibers and the matrix under high strain rates. This reduction in friction limits the fibers’ effectiveness in bridging multiple cracks, as supported by prior studies [30].

Figure 8a–c reveal that higher strain rates increase peak and ultimate strains in RPECC. For instance, C-20 exhibits a peak strain rise from 0.011 to 0.017 and an ultimate strain increase from 0.0278 to 0.0299 as strain rates escalate from 60.2 s^−1^ to 110.6 s^−1^. This strain-rate sensitivity is linked to the internal structural evolution of RPECC, where greater rubber content introduces soft particles that enlarge pore spaces and weaken interfacial bonds with the cementitious matrix [31]. This transformation enhances deformability under compression, reducing peak stress while increasing strain capacity. Additionally, RPECC subjected to thermal cycles presents trapezoidal stress–strain curves at strain rates surpassing 70 s^−1^, attributed to expanded porosity and interface degradation from repeated heating and cooling cycles. This accumulated internal damage diminishes impact resistance, as impact toughness calculations show untreated specimens outperform thermally cycled ones in energy absorption. The energy absorption capacity, represented by the area under the stress–strain curve, grows with strain rate, underscoring improved mechanical adaptability under complex stresses. Thus, the interplay of material composition and environmental factors defines RPECC’s operational limits in demanding applications.

#### 3.2.3. Dynamic Compressive Strength

The two-wave method was employed to analyze the experimental data, clarifying the relationship between dynamic compressive strength and the dynamic increase factor (DIF) under varying strain rates. According to reference [32], the DIF is defined as the ratio of dynamic strength to quasi-static strength. Table 4 shows that the dynamic compressive strength of the RPECC increases with rising strain rates, indicating their similar strain-rate sensitivity to that of ordinary concrete, consistent with previous studies [33,34,35,36]. As the rubber content gradually increases from 10% to 30%, the strengthening effect of the RPECC under impact loading diminishes. This observation implies that the overall dynamic strength enhancement increases and then decreases as rubber content increases, suggesting that there is an optimal rubber content (20%) that effectively enhances the strain-rate sensitivity of RPECC. However, exceeding a rubber content of 30% may negatively impact the dynamic compressive strength of the RPECC.

**Figure 9:**
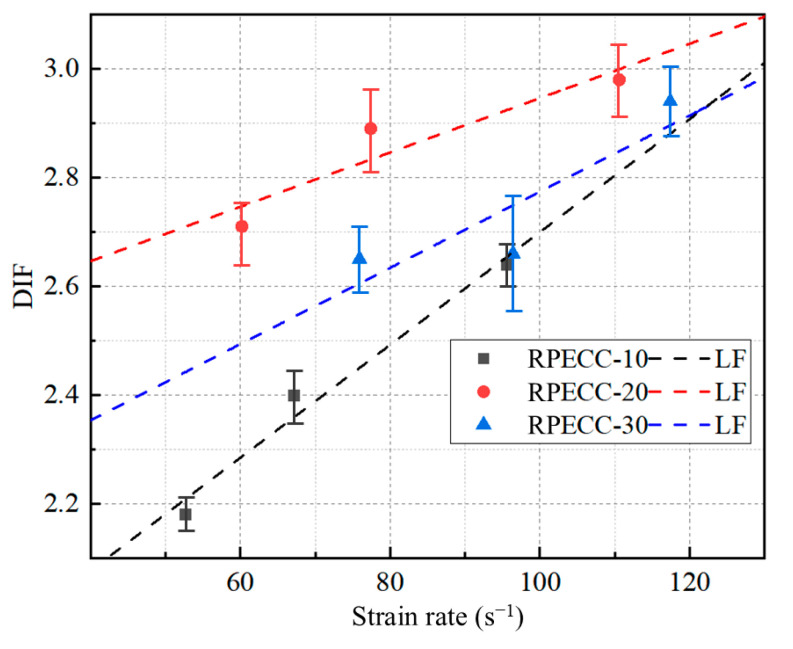
Relationship between strain rate and DIF.

Referring to specimen C-10, Table 4 reveals that increasing the strain rate from 52.6 s^−1^ to 67.1 s^−1^, and subsequently to 95 s^−1^, results in a dynamic compressive strength increase of 10% and 20.9%, respectively. The underlying reason is that a higher strain rate shortens the duration of the impact load on the specimen, resulting in transient effects. This phenomenon means that more energy is directed towards elevating the stress levels instead of generating and propagating internal cracks, thus contributing to enhanced dynamic compressive strength [37,38]. In comparison, for C-20, the same range of strain-rate changes yields increases of 6.9% and 9.9% in dynamic compressive strength. C-30 shows a more modest response, with a 1.0% increase initially, followed by a rise of 11.0%. Compared to Ref. [39]’s sisal fiber-reinforced concrete (36.8% tensile strength increase), RPECC’s dynamic strengthening (DIF = 2.64 for 10% rubber) relies more on rubber’s viscoelastic energy dissipation than fiber bridging. However, excessive rubber (30%) reduces DIF to 2.33, mirroring Ref. [40]’s finding that over 5% NGPs degrade workability due to agglomeration—both highlighting the criticality of optimal additive content for strain rate sensitivity. These findings suggest high rubber content can harm the material’s strain-rate strengthening capacity. Moreover, Figure 9 shows that the DIF displays a linear relationship with the strain rate, consistent with the findings of Kay M. F. et al. [41]. The DIF values for RPECC with varying rubbers increase with rising strain rates. Specifically, the DIF value for C-20 is generally higher than that of C-30, although the slopes of their linear relationships are comparable. In contrast, C-10 exhibits the steepest slope in its linear relationship, indicating that it has the greatest sensitivity to strain rate.

#### 3.2.4. Dynamic Peak Stress

Table 5 comprehensively summarizes the dynamic response results of RPECC under high-impact strain rates following thermal cycling. An observable trend emerges with an increase in the number of thermal cycles, where the dynamic peak stress of RPECC exhibits a gradual decline. This decline is distinctly convicted through the comparative analysis of the peak stresses for C-0-10, C-90-10, C-180-10, and C-270-10. Under strain rates of 95.6 s^−1^ and 114.3 s^−1^, a significant reduction in peak stresses is observed: C-90-10, C-180-10, and C-270-10 show decreases of 1.0%, 9.3%, and 15.1%, respectively, about C-0-10. Similar changes are evident across other specimens, potentially attributable to the progressive formation of pores on the specimen surfaces, which contributes to a looser and more porous material structure, thereby diminishing overall strength.

**Figure 10:**
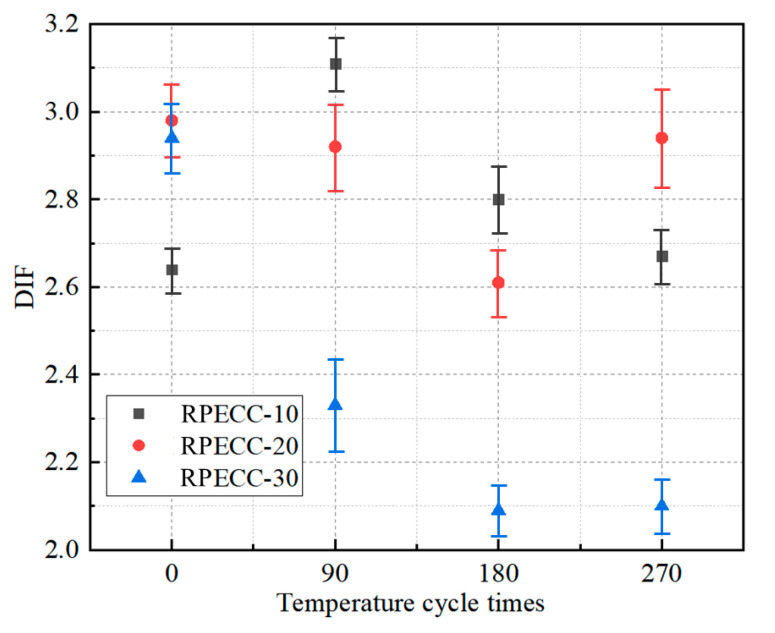
Relationship between cycle times and DIF.

Further insights reveal that under high-strain-rate conditions, the strength of the C-30 exhibits a significant decrease of 30.4% after 90 thermal cycles, while the C-20 and C-10 demonstrate reductions of 11.5% and 1.0%, respectively. This disparity suggests thermal cycle impacts are more pronounced in RPECC with elevated rubber content. The excessive incorporation of rubber may compromise the mechanical interlocking between aggregates, leading to a deterioration of the mechanical properties [42]. As the thermal cycles progress from 90 to 180 and then to 270, the dynamic compressive strength of the specimens exhibits reductions of 14.2% and 15.5%, respectively, indicating a diminishing rate of decline. This observation is attributed to the fact that thermal cycling induces differential expansion between the rubber and the cement matrix, which leads to interfacial microcracks and reduces the efficiency of stress transfer at high strain rates. Additionally, as shown in Figure 10, all specimens demonstrate a consistent downward trend in DIF values as the number of thermal cycles increases.

### 3.3. Microstructural Evolution

#### 3.3.1. Microscopic Analysis of RPECC

**Figure 11:**
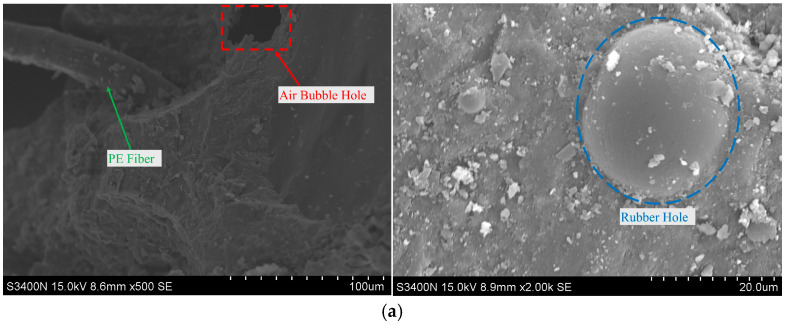

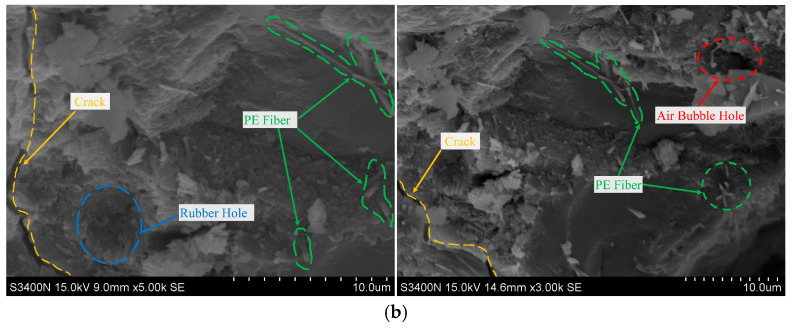
Internal structure of RPECC: (**a**) C-10; (**b**) C-30.

Figure 11 illustrates the micro-morphological characteristics of small fragments from C-10 and C-30 after impact damage, with images taken at various magnifications. These observations reveal the presence of sparse bubble defects and pores within the matrix, which are primarily linked to the incorporation of rubber particles. During the concrete mixing process, the addition of rubber particles disrupts chemical bonding, resulting in increased air content. Insufficient vibration during the pouring phase may also contribute to the entrapment of air bubbles. A higher rubber content is associated with a greater prevalence of pores and defects within the RPECC matrix. For example, due to its higher rubber content, C-30 displays a lower dynamic compressive strength compared to C-10. These findings demonstrate that rubber content significantly affects both the internal structure and mechanical properties of RPECC.

**Figure 12:**
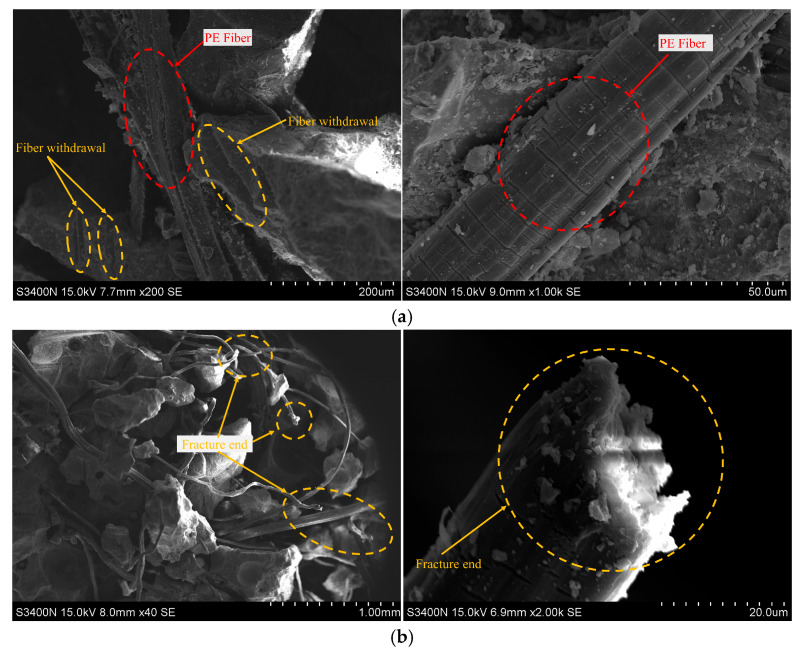
Failure modes of PE fibers in RPECC under dynamic loading, showing pull-out slip and fracture, with rubber incorporation promoting interfacial debonding: (**a**) Fiber withdrawal; (**b**) Fiber fracture.

Figure 12 provides a detailed examination of the microstructure of PE fibers subjected to high-strain-rate impact. Post-impact analysis indicates that the fibers remain relatively well-bonded to numerous fragmented substrates, facilitating two primary modes of micro-damage: fiber pull-out and fiber breakage. In Figure 12a, the PE fiber appears relatively flattened, exhibiting surface scratches of varying severity. The surrounding matrix shows clear indications of fiber pull-out, particularly in the region highlighted by the red circle, suggesting that the fiber has slipped into the matrix. In contrast, Figure 12b depicts a scenario where one end of the fiber is forcibly “pulled” while the other remains embedded, indicating fiber breakage. This “pull-off” phenomenon reveals fibers’ critical role in load transfer during impact events. However, under thermal conditions, the load-transfer efficiency of the fibers may be compromised, leading to significant deformation in a relatively short timeframe and potential detachment. Furthermore, fiber pull-out predominantly occurs in regions where fibers are bonded with rubber particles, as the rubber functions merely as filler within the matrix and fails to establish a mature fiber-bridging structure through hydration, thereby rendering fiber–rubber bonds more susceptible to failure.

#### 3.3.2. Energy-Dispersive X-Ray Spectroscopy (EDS) for Microstructural Analysis

**Figure 13:**
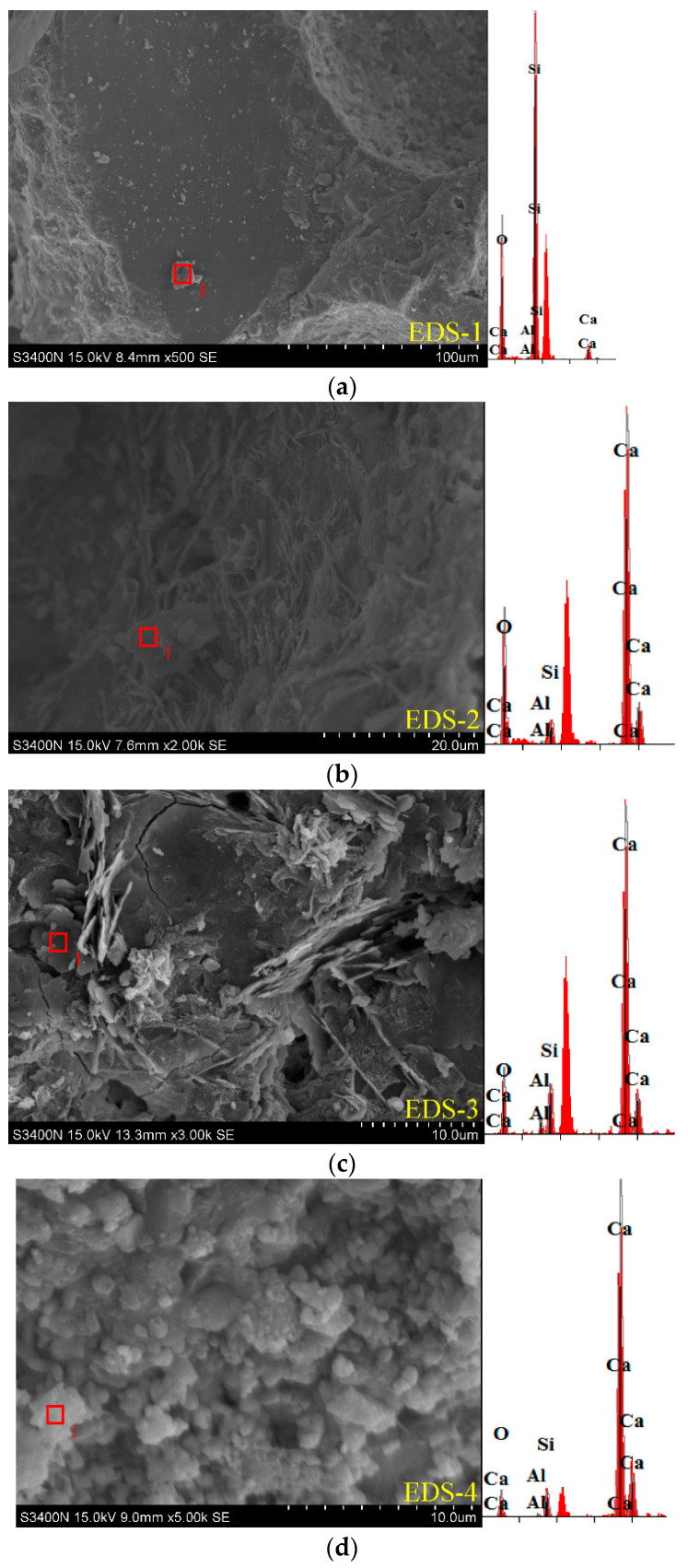
Microtopography images and EDS: (**a**) C-0-20; (**b**) C-90-20; (**c**) C-180-20; (**d**) C-270-20.

Figure 13 presents the micromorphology of the internal hydration products of RPECC subjected to varying numbers of thermal cycles. Table 6 summarizes the elemental content of the internal hydration products of RPECC. These observations were conducted using a high-power microscope with EDS analysis. As the number of thermal cycles increases from 90 to 270, a significant transformation occurs in the internal structure of RPECC. The structure evolves from a dense, layered configuration characterized by a limited presence of acicular crystals to a more square-shaped and flocculent morphology. The predominant elements in the flake-shaped, block-shaped, and flocculent crystals include silicon (Si), oxygen (O), aluminum (Al), and calcium (Ca). It is hypothesized that these crystals may correspond to calcium silicate hydrate (C-S-H) gel, calcium hydroxide (CH), or ettringite (AFt). As shown in Table 6, further analysis of calcium and oxygen content suggests that the initial internal composition of RPECC (C-0-20) is primarily comprised of dense C-S-H gel, evidenced by a Ca/Si ratio of ~1.6 (characteristic of stable hydration products). However, as thermal cycles increase, ongoing hydration reactions and thermal stress accelerate the decomposition of C-S-H, gradually forming low-strength CH. Quantitative analysis reveals a critical shift: the Ca/Si ratio surges from ~1.6 in C-0-20 to >20 in C-270-20, accompanied by a 33-fold increase in calcium content and a 92% decrease in silicon content. This directly indicates progressive C-S-H degradation and CH precipitation.

In contrast to Ref. [40]’s nano graphite platelets (NGPs), which reduce porosity by 32.2% through a filler effect to densify the matrix, or Ref. [39]’s Bacillus subtilis-induced calcite precipitation that strengthens fiber matrix interfaces, thermal cycling in this study accelerates the formation of weak calcium hydroxide (CH). The Ca content increase from 2.23% to 74.09% in Table 6 mirrors the uncontrolled hydration defects in Ref. [39]’s control mix, where lack of bacterial reinforcement leads to durability degradation. Additionally, the rubber matrix interface degradation observed in SEM (Figure 11b)—characterized by microcrack propagation—mimics the fiber matrix debonding seen in Ref. [39]’s mixes without bacterial agents, both highlighting the critical role of interfacial integrity in resisting thermal damage.

Rubber particles exacerbate this trend: high rubber content (30%) in C-30 (Figure 11b) introduces interfacial voids and hinders C-S-H gel formation, while PE fibers in C-20 (Figure 13b) mitigate CH growth by promoting uniform hydration. This aligns with Ref. [43]’s finding that hybrid fibers enhance matrix densification by reducing CH orientation and improving C-S-H connectivity, thereby sustaining strength under thermal stress.

Unlike C-S-H’s amorphous, load-distributing structure, CH’s plate-like crystals create stress concentrations at the matrix-fiber interface, weakening mechanical integrity. The emergence of these calcium hydroxides contributes to the development of weak points within the internal matrix, correlating with the 18.0% decline in static compressive strength observed after 270 cycles (Section 3.1.3). These analytical outcomes underscore the significant impact of thermal cycles on the hydration products of RPECC, which subsequently influences its macroscopic mechanical properties.

## 4. Conclusions

This study experimentally investigates the static and dynamic compressive mechanical properties of RPECC following thermal-cycle treatment. A comprehensive analysis of the experimental results leads to several key conclusions:

1. Failure mode of RPECC resembles that of rubber concrete, with damage mainly in peripheral areas. Larger fragments are held together by fibers, allowing some structural integrity under static compression. Under dynamic loads, increased strain rate, rubber particle content, and thermal-cycle frequency worsen the damage, showing the material’s high sensitivity to external conditions.
2. Thermal cycles significantly reduce the compressive strength of RPECC, with a pronounced decline as cycles increase. After 270 cycles, static compressive strength drops by 18.0%, while dynamic compressive strength falls by 41.2%. The dynamic strength of RPECC rises with higher strain rates, demonstrating a strain-rate sensitivity similar to that of conventional concrete.
3. Adding rubber to RPECC reduces static compressive strength, decreasing by 17.9% at 30% rubber content and by only 11.3% at 20% rubber content. However, an optimal rubber proportion (20%) exists for enhancing strain-rate sensitivity under dynamic loading. Beyond this threshold, the material’s dynamic performance may deteriorate. Moreover, the stress–strain response of the 20% rubber specimens is more elastic with higher peak strain values, achieving a balance between ductility and strength loss.
4. SEM images indicate that PE fibers primarily fail through fracture and pull-out slip, with the addition of rubber shifting the failure mechanism towards pull-out slip. EDS analysis shows that thermal fatigue and hydration promote the formation of hydration products, especially calcium hydroxide (CH), which significantly reduces the dynamic compressive strength of RPECC after thermal cycles. Furthermore, thermal cycling affects RPECC properties in two ways: (i) the CTE difference between rubber and matrix triggers interfacial microcracks, and high temperature accelerates the aging of rubber, weakening its stress dispersion ability; (ii) the interfacial bond between PE fibers and matrix is degraded by thermal cycling, and the fiber-bridging effect is reduced, which, together with the formation of the hydration product CH, results in the deterioration of the mechanical properties.

## Figures and Tables

**Table 1:** Mix proportions of RPECC.

Code	Cement	Fly Ash	Blast Furnace Slag	Sand	Water	Superplasticizer /%	PE Fiber /%	Cycles	Rubber Content /%
C-0-10	0.75	0.1	0.15	0.45	0.25	1.0	2	0	10
C-0-20				0.40					20
C-0-30				0.35					30
C-90-10				0.45				90	10
C-90-20				0.40					20
C-90-30				0.35					30
C-180-10				0.45				180	10
C-180-20				0.40					20
C-180-30				0.35					30
C-270-10				0.45				270	10
C-270-20				0.40					20
C-270-30				0.35					30

Note: taking “C-0-10” as an example, “C” means RPECC, “0” means that the number of thermal cycles is 0, and “10” means that the rubber particle content is 10% (volume fraction).

**Table 2:** Properties of PE fibers.

Length/mm	Diameter/µm	Elongation at Break/%	Tensile modulus/MPa	Tensile Strength/MPa	Density/(g/cm^3^)
18	24	2	116 × 10^3^	3000	0.97–0.98

**Table 3:** Quasi-static compression performance.

Specimen Code		Peak Stress/MPa	Average Value/MPa	Standard Deviation	Modulus of Elasticity/GPa	Average Value/GPa	Standard Deviation
C-0-10	a	52.10	50.01	2.16	25.01	24.61	0.41
	b	47.91			24.20		
C-0-20	a	44.54	44.37	0.67	23.11	22.97	0.14
	b	43.20			22.83		
C-0-30	a	41.10	42.11	1.01	21.73	21.87	0.14
	b	43.12			22.01		
C-90-10	a	43.11	42.06	1.05	22.81	22.54	0.27
	b	41.01			22.27		
C-90-20	a	40.34	40.05	0.29	22.19	21.92	0.27
	b	39.76			21.65		
C-90-30	a	38.14	37.00	1.14	21.10	20.79	0.31
	b	35.86			20.48		
C-180-10	a	43.39	42.83	0.56	21.80	21.61	0.19
	b	42.27			21.42		
C-180-20	a	39.79	40.41	0.62	21.39	21.63	0.24
	b	41.03			21.87		
C-180-30	a	34.95	35.39	0.44	20.81	20.56	0.25
	b	35.83			20.31		
C-270-10	a	42.61	42.03	0.58	21.49	21.30	0.19
	b	41.45			21.11		
C-270-20	a	40.61	39.38	1.12	21.09	20.81	0.28
	b	38.38			20.53		
C-270-30	a	34.85	34.52	0.33	19.79	19.90	0.11
	b	34.19			20.01		

Note: taking “C-0-10” as an example, “C” means RPECC, “0” means that the number of thermal cycles is 0, “10” means that the rubber particle content is 10% (volume fraction), and so on.

**Table 4:** Dynamic compressive performance under different strain rates.

Specimen Code	Dynamic Compressive Strength/MPa	Strain Rate/s^−1^	DIF	Dynamic Toughness/(MJ/m^3^)	Energy Absorption Rate Strength/%
C-10-0.6	109.18	52.6	2.18	1.11	1.017
C-10-0.7	120.13	67.1	2.40	1.46	1.215
C-10-0.8	132.02	95.6	2.64	2.45	1.856
C-20-0.65	120.13	60.2	2.71	1.46	1.215
C-20-0.7	128.37	77.4	2.89	1.29	1.005
C-20-0.8	132.03	110.6	2.98	2.01	1.522
C-30-0.6	111.40	75.9	2.65	2.08	1.867
C-30-0.7	112.04	96.4	2.66	1.86	1.660
C-30-0.8	123.62	117.4	2.94	2.21	1.788

Note: taking “C-10-0.6” as an example, “C” means RPECC, “10” means that the rubber particle content is 10% (volume fraction), “0.6” corresponds to an impact air pressure of 0.6 MPa, and so on.

**Table 5:** Dynamic compressive properties at high strain rate.

Specimen Code	Dynamic Compressive Strength/MPa	Strain Rate/s^−1^	DIF	Dynamic Toughness/(MJ/m^3^)	Energy Absorption Rate Strength/%
C-0-10	132.02	95.6	2.64	2.45	1.856
C-0-20	132.03	110.6	2.98	2.01	1.522
C-0-30	123.62	117.4	2.94	2.21	1.788
C-90-10	130.66	109.6	3.11	1.45	1.110
C-90-20	116.80	113.6	2.92	1.81	1.550
C-90-30	86.03	131.6	2.33	1.14	1.325
C-180-10	119.76	106.1	2.80	2.03	1.695
C-180-20	105.43	120.3	2.61	1.16	1.100
C-180-30	73.85	141.1	2.09	0.93	1.259
C-270-10	112.11	114.3	2.67	1.93	1.722
C-270-20	115.73	124.1	2.94	1.75	1.512
C-270-30	72.66	129.2	2.10	0.96	1.321

Note: taking “C-0-10” as an example, “C” means RPECC, “0” means that the number of thermal cycles is 0, “10” means that the rubber particle content is 10% (volume fraction), and so on.

**Table 6:** Elemental content of internal hydration products of RPECC.

Specimen Code	Element Content/%			
	O	Al	Si	Ca
C-0-20	61.883	0.287	35.599	2.231
C-90-20	49.635	0.302	1.922	48.141
C-180-20	35.598	1.500	4.820	58.083
C-270-20	22.589	0.366	2.956	74.090

## Data Availability

The original contributions presented in this study are included in the article material. Further inquiries can be directed to the corresponding author.
